# Presence of Three-Dimensional Sound Field Facilitates Listeners’ Mood, Felt Emotion, and Respiration Rate When Listening to Music

**DOI:** 10.3389/fpsyg.2021.650777

**Published:** 2021-11-17

**Authors:** Yuuki Ooishi, Maori Kobayashi, Makio Kashino, Kanako Ueno

**Affiliations:** ^1^NTT Communication Science Laboratories, NTT Corporation, Atsugi, Japan; ^2^Faculty of Human Sciences, School of Human Sciences, Waseda University, Tokorozawa, Japan; ^3^Department of Architecture, School of Science and Technology, Meiji University, Kawasaki, Japan; ^4^Core Research for Evolutional Science and Technology, Japan Science and Technology Agency (CREST, JST), Tokyo, Japan

**Keywords:** music, autonomic nerve, sound field, three dimension (3D), respiration, mood, emotion

## Abstract

Many studies have investigated the effects of music listening from the viewpoint of music features such as tempo or key by measuring psychological or psychophysiological responses. In addition, technologies for three-dimensional sound field (3D-SF) reproduction and binaural recording have been developed to induce a realistic sensation of sound. However, it is still unclear whether music listened to in the presence of 3D-SF is more impressive than in the absence of it. We hypothesized that the presence of a 3D-SF when listening to music facilitates listeners’ moods, emotions for music, and physiological activities such as respiration rate. Here, we examined this hypothesis by evaluating differences between a reproduction condition with headphones (HD condition) and one with a 3D-SF reproduction system (3D-SF condition). We used a 3D-SF reproduction system based on the boundary surface control principle (BoSC system) to reproduce a sound field of music in the 3D-SF condition. Music in the 3D-SF condition was binaurally recorded through a dummy head in the BoSC reproduction room and reproduced with headphones in the HD condition. Therefore, music in the HD condition was auditorily as rich in information as that in the 3D-SF condition, but the 3D-sound field surrounding listeners was absent. We measured the respiration rate and heart rate of participants listening to acousmonium and pipe organ music. The participants rated their felt moods before and after they listened to music, and after they listened, they also rated their felt emotion. We found that the increase in respiration rate, the degree of decrease in well-being, and unpleasantness for both pieces in the 3D-SF condition were greater than in the HD condition. These results suggest that the presence of 3D-SF enhances changes in mood, felt emotion for music, and respiration rate when listening to music.

## Introduction

Listening to music can elicit many kinds of emotional responses in humans (Chanda and Levitin, 2013; Koelsch, 2014). Psychological studies have revealed that music listening induces emotions and changes in moods (Blood et al., 1999; Nayak et al., 2000; Murrock and Higgins, 2009; Koelsch and Jancke, 2015). Psychophysiological studies have revealed that music-induced emotions are strongly associated with the modulation of the physiological system, which typically includes changes in heart rate, heart rate variability (HRV), and respiration rate. Listening to self-selected music that can cause chills induces increases in heart rate and respiration rate (Salimpoor et al., 2009, 2011). Listening to relaxing music suppresses stress-induced increases in heart rate (Knight and Rickard, 2001) and increases the amplitude of the high-frequency (HF) component of HRV, which is an index of parasympathetic nerve activity (Zhou et al., 2010). A similar symmetry property of music in physiological activities has been observed on other emotional axes such as happiness and sadness. An earlier study demonstrated that happy and sad excerpts can be distinguished with respiration rate (Etzel et al., 2006).

In investigations of the mechanisms that underlie music-induced emotion or its related physiological activities, musical features have been considered factors determining the effects of music listening on humans. For example, tempo has been considered a factor determining whether the effect of listening to music is exciting or relaxing. Listening to music with a fast tempo causes an increase in sympathetic nerve activity (Bernardi et al., 2006), while music with slow tempos reduces heart rate and is felt to be relaxing (Standley, 1986). The happy-sad music distinction depends on the combination of tempo and key (Hevner, 1935; Balkwill and Thompson, 1999). Gomez and Danuser (2007) have summarized the effects of several music features, such as intensity, tempo, rhythm, and accentuation, on the levels of arousal and valence or physiological activities.

However, the effect of being surrounded by three-dimensional (3D) sounds when listening to music has not been clarified. As represented by multichannel reproduction systems such as 22.2-channel sound systems, based on two-channel stereo (Hamasaki et al., 2008) or wave field synthesis techniques (Berkhout et al., 1993), technology for reproducing a 3D-sound field (3D-SF) has been developed to achieve a realistic sensation of sound, namely a sense of an acoustic field, which means that we feel as if we are in a real sound field, such as that of a live music performance. Developers of 3D-SF reproduction techniques have thought that being surrounded by 3D sounds is an important factor in music listening (Chen, 2001; Fukada, 2001). Actually, the presence of a 3D-SF enhances self-reported impressions of sound (e.g., presence, naturalness, brightness, reality, or beauty) compared with normal stereophonic sound (Hamasaki et al., 2008). In the sense of realizing a feeling of a sound field, however, binaurally recorded sounds also enable listeners to feel as if they were in the original sound field (Paul, 2009). Self-reported impressions of sound are higher when binaurally sounds recorded with a dummy head are reproduced through headphones than they are for normal stereophonic sounds through headphones (Paul, 2009). Both 3D-SF reproduction and binaural recording techniques have aimed at inducing a sense of sound realness, and the spatial audio information is almost the same between them. However, we believe that there are significant differences in psychological perception or physiological responses between a reproduced 3D-SF and binaurally recorded sound. Since our body is exposed to vibrations of air when we listen to sounds in a physically existing 3D-SF, we hypothesized that when we are exposed to music in the presence of a physical 3D-SF, the physical 3D-SF conveys information not only through auditory perception but also through other channels, such as tactile ones evoked by the effect of music-induced air vibration on the body of a listener, which might affect the psychological and physiological states of listeners. A couple of studies have indicated interactions between auditory and tactile information. One showed that musical meter recognition by musicians for auditory stimulation is disturbed with the presence of incongruent tactile stimulations (Huang et al., 2012). Another found that musicians have a higher ability of frequency discrimination in not only auditory but also tactile stimulation than non-musicians (Sharp et al., 2019). Since these two studies presented tactile stimulations on the fingertip or hand of participants independent of auditory stimulations, they did not examine the tactile sensation evoked by the air vibration that sound stimulations induce. Earlier studies showed the impact of listening to live musical performances. For example, live music was found to relieve tension-anxiety and enhance the vigor of patients with cancer compared with tape-recorded music (Bailey, 1983). More recent studies have discussed the effect of live music from the viewpoint of audiovisual interaction. The combination of audition and vision boosts the reward functions of an audience with higher electrodermal activities than when they are exposed to an audition-alone or vision-alone condition when listening to music (Chapados and Levitin, 2008). Other studies have shown the effects of the presence of other people at live music performances, including audiences and performers. The spontaneous presence of others has an influence on the reduction in cardiovascular reactivity to mental stress (Phillips et al., 2006). Sharing musical moments with lively performing musicians enhances the parasympathetic nerve activity more efficiently than when listening to recorded music, suggesting that not only verbal but also non-verbal communication occurs between the audience and performer (Shoda et al., 2016). However, these studies examining the effects of listening to live music have never assessed the potential effects of a physically existing 3D-SF on listeners of live music.

In this study, we examined whether the presence of a physical 3D-SF of music accelerates psychological and physiological responses when listening to music. To do this, we used a novel 3D-sound reproduction system (Omoto et al., 2015) and compared the effect of music reproduced through this system with that through headphones. Previous studies have measured mood (Nayak et al., 2000; Murrock and Higgins, 2009) and pleasantness or unpleasantness (Blood et al., 1999; Koelsch and Jancke, 2015) as psychological indices, and respiration rate and cardiac autonomic responses (Bernardi et al., 2006; Salimpoor et al., 2011) as physiological indices to examine the effects of music listening. Accordingly, this study also measured these indices.

## Materials and Methods

### Participants

Thirty-two healthy students of Meiji University (age 22.5 ± 0.4 (SEM) years old, 3 females) participated in the experiment. None of them were musicians, and they did not have any musical training other than basic music courses in primary and secondary education. All procedures were conducted in accordance with the Declaration of Helsinki and approved by the Ethics and Safety Committees of Meiji University, and the participants were paid for their participation.

### Music Stimuli and Listening Environment

**FIGURE 1:**
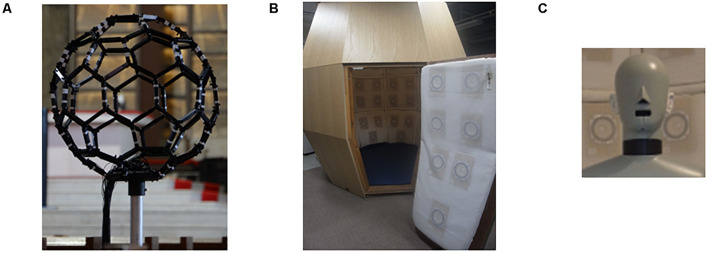
Experimental equipment. **(A)** Photograph of fullerene-like microphone array with 80 channels of the BoSC system. **(B)** Photograph of reproduction system with 96 loudspeakers of the BoSC system. **(C)** Photograph of dummy head that had microphones in the position of the eardrums.

We selected two music genres for the experimental music stimuli: pipe organ music (PM) and acousmonium (AC). They were chosen because they both have rich echo, so listeners can effectively receive music-induced air vibration from all directions and feel as if they were surrounded by music. For the preparation of PM, a Japanese professional musician played Toccata and Fugue in D minor BWV 565 by Bach with a pipe organ in a church, which was recorded live using a fullerene-like microphone array^1^ (Figure 1A). A piece of AC was composed by a Japanese musician, and he/she played it him/herself in a multipurpose hall with multiple speakers, which was also recorded live^2^. The lengths were 543 s for PM and 563 s for AC. The participants were very familiar with the introductory segment of Toccata and Fugue, but none of them had ever listened to it all the way through. They did not know the AC piece.

To elucidate the effect of the sound field of music, two types of sound reproduction conditions were prepared, namely a 3D-SF condition and an HD condition. We used the different sound presentation systems for each condition as follows.

### Three-Dimensional Sound Field Condition

**FIGURE 2:**
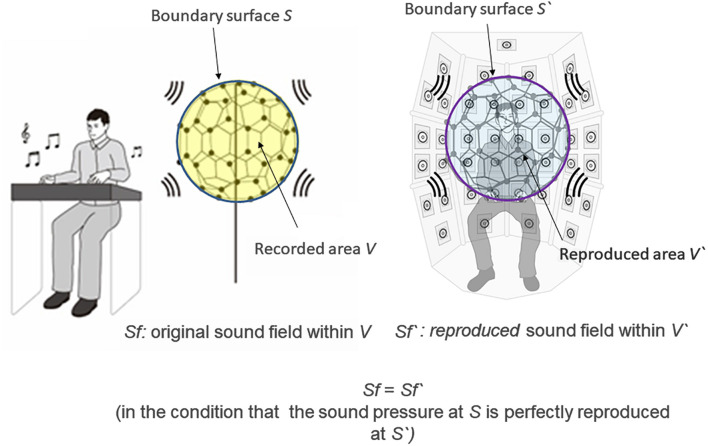
Scheme of the boundary surface control (BoSC) principle. Left: Original sound was recorded with a microphone array. *Sf* stands for the original sound field within the enclosed space of the microphone array (defined as recorded area *V*), and *S* stands for the boundary surface of the recorded area *V*. Right: Recorded original sound was reproduced in a reproduction room. We imagine that there was the same microphone array as in the left panel at the listening position, and let *Sf′* stand for the reproduced sound field within the enclosed space of the virtual microphone array (defined as reproduced area *V′*) and let *S′* stand for the boundary surface of the reproduced area *V′*. If the original sound pressure at *S* is perfectly reproduced at *S′*, *Sf′* is mathematically completely the same as *Sf*.

**FIGURE 3:**
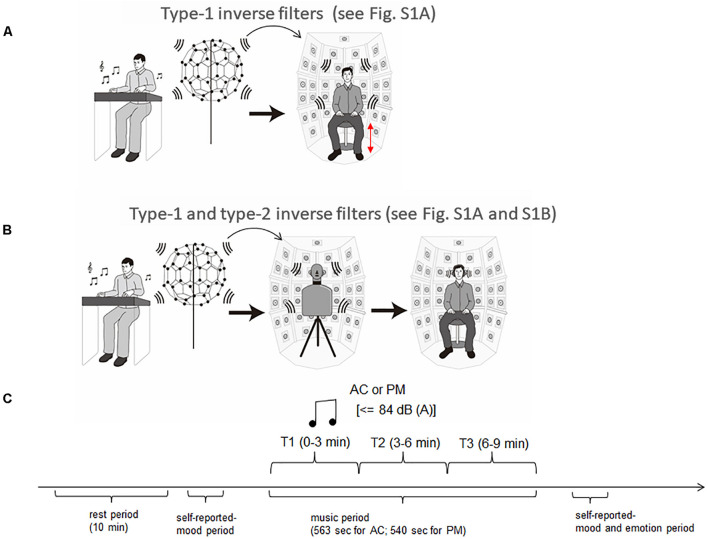
Experimental setup and procedure. **(A)** Music presentation in the three-dimensional sound field (3D-SF) condition. Music was recorded with the fullerene-like microphone array with 80 channels, and was reproduced in the BoSC reproduction room with 96 channels. To reproduce the accurate sound field in the listening area, type 1 inverse filters were convolved with recorded music signals. The height of the chair was adjusted for each participant so that his/her head position was completely enclosed within the fullerene-like microphone array, which was used for the preparation of type 1 inverse filters. **(B)** Music presentation in the HD condition. Music in the 3D-SF condition was recorded in the Sound Cask through a dummy head and reproduced with headphones. To remove the frequency characteristics of the headphones, type 2 inverse filters were convolved with the music signals used in the 3D-SF condition. **(C)** Experimental procedure. Participants experienced one 10-min period of silence as a rest period. After that, they reported their mood and entered the session of music listening. When they finished the listening session, they performed a second self-report of mood and assessed the felt emotion of the music they had listened to.

In the 3D-SF condition, we used a 3D-SF reproduction system based on the boundary surface control (BoSC) principle (Ise, 1999), the details of which have been previously reported (Enomoto et al., 2011; Omoto et al., 2015). A 3D-SF reproduction system based on this principle is called a BoSC system (Enomoto et al., 2011). This system comprises a microphone array and a reproduction room. We used a fullerene-like microphone array consisting of 80 omnidirectional microphones (4060; DPA Microphones, Lillerød, Denmark), each of which was located in the position of the carbon molecule in C80 fullerene (Figure 1A). Music was recorded with the microphone array at a sampling rate of 48 kHz, and we used 24-bit quantization. Recorded music pieces were reproduced in the reproduction room, which contained seven-layer loudspeaker arrays based on height, forming what is called the Sound Cask (Figure 1B). Six loudspeakers (FE83E; Fostex, Tokyo, Japan) were installed on the ceiling plane. The other speakers were installed on surface of the wall at six different heights. Nine loudspeakers were installed at the highest and lowest points, and 18 were allotted to each of the remaining heights. The system accommodated 96 loudspeakers in total (Figure 1B). The BoSC principle supports the following idea (see Figure 2). An original sound is recorded with a microphone array in an enclosed area. This enclosed area is defined as recorded area *V*, the boundary surface enveloping recorded area *V* is defined as *S*, and the sound field within recorded area *V* is defined as *Sf* (Figure 2, left). If the recorded original sound is reproduced in a reproduction room, and the sound field within the reproduced area *V′*, which has completely the same shape and size as recorded area *V*, is defined as *Sf*′, then *Sf′* is mathematically equal to *Sf* under the condition that the sound pressure at the boundary surface *S* is perfectly reproduced at the boundary surface *S′*, which envelopes the reproduced area *V′* (Figure 2, right) (Ise, 1999; Omoto et al., 2015). To best approximate this with our speakers, we set the fullerene-like microphone array in the listening position in the Sound Cask and recorded time stretched pulse (TSP) signals reproduced from the speakers. From the recorded sound data, we calculated the transfer function *T*_*ij*_ between each speaker *SP*_*i*_ (i = 1, 2, 3,…96) and each microphone *MC*_*j*_ (j = 1, 2, 3,…80), and we prepared inverse filters (type-1 inverse filters) that consisted of the inverse transfer function *invT*_*ij*_, by which the transfer function *T*_*ij*_ between speakers and microphones can be cancelled (Enomoto et al., 2011) (Supplementary Figure 1A). We convolved the type-1 inverse filters to recorded music stimuli. By these processes, we were able to present the spatial information of the original sound field to listeners with state-of-the-art approximation, and the listeners were to experience the original sound field as if they existed in it (Figure 3A).

### HD Condition

In the HD condition, the same convolved music signals as in the 3D-SF condition were recorded in the Sound Cask once using a dummy head that has microphones in the position of the ear drums (HATS 4128C; Bruel & Kjaer, Nærum, Denmark; Figure 1C) to perform binaural recording. The sampling rate was 48 kHz, and we used 24-bit quantization as we did in the 3D-SF condition. The participants listened to the music signals through headphones (HDA 300; Sennheiser, Wedemark, Germany) in the listening area of the Sound Cask. To correct the music signals according to the frequency response of the headphones, like we did in 3D-SF condition, we prepared type 2 inverse filters that removed the frequency characteristics of the headphones (see Supplementary Figure 1B). With such a procedure, the participants in the HD condition were able to experience the spatial-audio information of music as rich as that in the 3D-SF condition (Figure 3B). The difference between the two conditions was the presence of the physical 3D-SF as the vibration of air around the listeners or not. If the participants were affected by the presence of the physical 3D-SF through any channels other than auditory perception, such as tactile ones, we expected to observe psychological or physiological differences between the 3D-SF and HD conditions.

### Self-Reported Moods and Emotions

The method of multiple mood scaling (Terasaki et al., 1992) was used to examine the influence of music on the mood of the participants. We evaluated the strength of the following classified moods: Boredom, Liveliness, Well-being, Friendliness, Concentration, and Startle, each of which consisted of 10 mood-related adjectives such as tranquil or cautious, 60 adjectives in total. All the mood-related adjectives were written in Japanese. Before and after they had listened to music, the participants were asked to report the intensity in which they felt each mood-related adjective on a 4-point scale: 1. “don’t feel at all”; 2. “don’t feel so much”; 3. “feel to some extent”; and 4. “feel very much.” The order of the adjectives was completely randomized for each participant. After the second rating of mood-related adjectives, the participants were asked to judge the emotions they felt from the music with “pleasant” and “unpleasant.” After the judgment, they rated the degree of felt emotion for the music they had listened to on a 10-point scale: pleasant (1 = not pleasant at all, 10 = very pleasant); unpleasant (1 = not unpleasant at all, 10 = very unpleasant). When a participant selected one of “pleasant” or “unpleasant” and rated the score from 1 to 10, the score of the other was automatically 1.

### Physiological Measurements

Electrocardiograms and an elastic chest band (Polyam-RESP, Nihon Santeku, Osaka, Japan) were used to measure interbeat intervals (R-R intervals) of the heart and respiration, respectively, throughout the experiments. Analogue data were amplified and digitized with BIOPAC MP150 (BIOPAC Systems, Goleta, United States). The sampling rate was 1,250 Hz for both the ECGs and respiration measurements.

To calculate respiration rate, raw respiration signals were filtered with a.05–0.5-Hz band pass filter. The peak detection was performed with AcqKnowledge (analysis software of the BIOPAC MP150, United States) from the filtered respiration signals, and peak-to-peak intervals were obtained. The collected peak-to-peak interval data were resampled to 10 Hz by linear interpolation. For the respiration rate analysis, the interpolated data were converted to second-by-second values and expressed in cycles per minute (cpm) by dividing each value by 60.

To calculate the R-R intervals in the ECG measurement, R-wave detection was performed again with AcqKnowledge, and the result was visually screened to eliminate any inappropriate R-wave detection related to artifacts such as movement. The appropriately collected R-R interval data were resampled to 10 Hz by cubic spline interpolation. For the heart rate analysis, the interpolated R-R interval data were converted to second-by-second values and expressed in beats per minute (bpm) by dividing each value by 60.

For the HRV analysis, a fast Fourier transform (FFT) was applied to the interpolated R-R interval data after removing the linear trend to calculate the HRV power spectra using a Hanning window. Low-frequency (LF) and high-frequency (HF) components were obtained by integrating the power spectra over their respective ranges of 0.04–0.15 and 0.15–0.4 Hz. The magnitude of the HF and the ratio of LF to HF (LF/HF) correspond to the strength of the vagal activity (Porges, 1995) and the sympathovagal balance (Task Force, 1996), respectively. The magnitude of the LF involves both vagal and sympathetic nerve activities (Pomeranz et al., 1985). FFT was applied to each 3-min window of the interpolated data series of R-R intervals. The magnitude of each spectral component was evaluated using the natural logarithms of the power (lnLF and lnHF). The ratio of the LF component to the HF component (LF/HF ratio) was evaluated by dividing lnLF by lnHF (lnLF/lnHF).

### Experimental Procedure

The 32 participants were divided into two groups: the 3D-SF condition group (16 members, one female) and the HD condition group (16 members, two females). They came to the laboratory for 2 days, and on each day, they listened to one of the music pieces (AC or PM). On the first day of the experiment, they were given general information about the experiment upon arrival, and their written consent was obtained. The experimental procedure consisted of four periods: rest period → self-reported-mood period→ music period → self-reported-mood and emotion period. In the Sound Cask, the participants were asked to keep their eyes open during the experiment, and to steadily sit on a chair and avoid moving any part of their body, such as the head, as much as they could. The Sound Cask was kept dark during the experiment. The height of the chair was adjusted for each participant so that the position of the head was completely enclosed within the fullerene-like microphone array, which was used for the preparation of type-1 inverse filters. The participants were outfitted with ECG transducer electrodes and an elastic chest band for 10 min to familiarize them with the experimental environment. The participants in the HD condition group wore headphones. This time period is referred to as the “rest period.” The last 3 min of the ECG and respiration recording was regarded as the baseline. Prior to the listening session, the participants were asked to rate the intensity of the 60 mood-related adjectives on a 4-point scale. When the listening session started (music period), the participants were presented with one of the music pieces with a maximum level of 84 dB SPL (A) for 540 s for PM and 563 s for AC. Then, there was a second period during which the participants were asked to rate the same 60 mood-related adjectives as in self-reported-mood period 1 and the felt emotion of this music on a 10-point scale (self-reported mood and emotion period). The order of the music pieces was completely randomized. The experimental procedure is summarized in Figure 3C.

### Data Analysis

Data are presented as means ± SEM, and a probability value *p* < 0.05 was considered to be statistically significant.

Time *t* = 0 was set as the onset of music. Physiological measures (respiration rate, heart rate, and HRV) were evaluated by averaging the data with a 3-min window (*t* = 0 to 3, *t* = 3 to 6, and *t* = 6 to 9 min for each) in the music period. The corresponding 3-min time regions were defined as T1, T2, and T3. To exclude the effect of the variation on the baseline value, the averaged data were normalized by dividing them by the basal value for each measurement in the baseline recording. The changes in these physiological measures normalized by the basal value were analyzed by three-factor repeated measures ANOVA with “sound reproduction condition” (3D-SF and HD) as a between-subjects factor and “music” (AC and PM) and “time” (baseline, T1, T2, and T3) as within-subjects factors.

The self-reported scores for the 60 mood-related adjectives were averaged among the corresponding 10 adjectives to determine the strength of each classified mood. The difference between the change in each mood (Boredom, Liveliness, Well-being, Friendliness, Concentration, and Startle) for different conditions was analyzed by two-factor repeated measures ANOVA with “sound reproduction condition” (3D-SF and HD) as a between-subjects factor and “music” (AC and PM) as a within-subjects factor.

Since all the participants rated their felt emotion as unpleasant for both AC and PM in both the 3D-SF and HD conditions, unpleasantness alone was analyzed by two-factor repeated measures ANOVA with “sound reproduction condition” (3D-SF and HD) as a between-subjects factor and “music” (AC and PM) as a within-subjects factor.

Multiple comparisons between the three time regions of the music period (T1, T2, and T3) and baseline were analyzed with Ryan’s method. For the repeated measures ANOVA, Huynh-Feldt corrections were applied when appropriate.

## Results

### Effect of Difference in Sound Reproduction Method on Self-Reported Moods and Emotions

**FIGURE 4:**
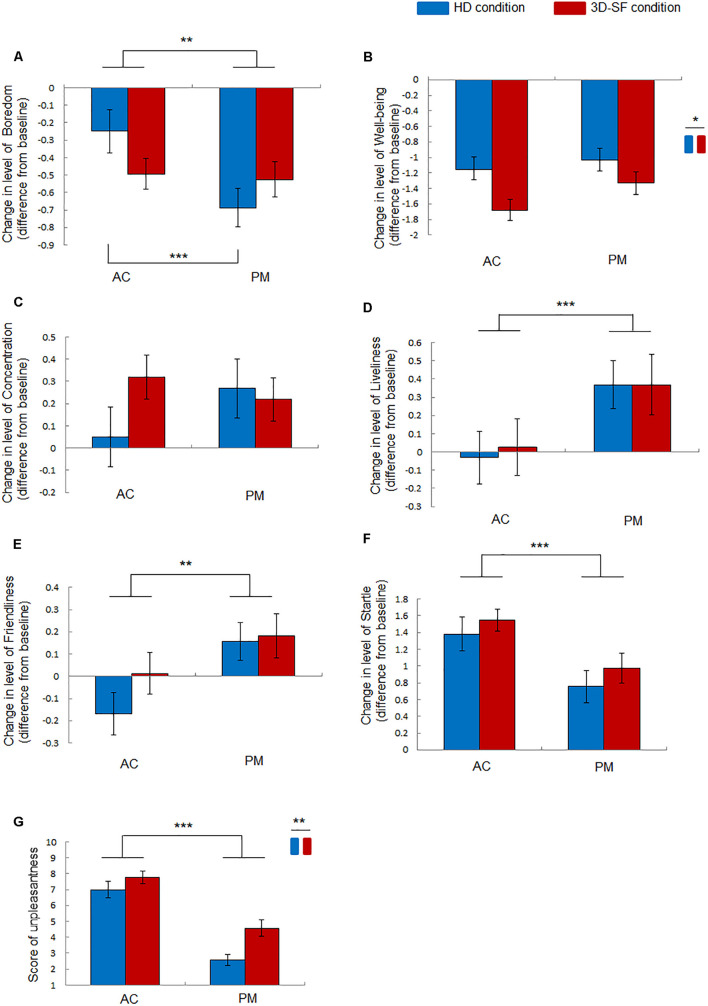
Effect of music listening on moods and unpleasantness of music. **(A–F)** Change in the level of **(A)** Boredom, **(B)** Well-Being, **(C)** Concentration, **(D)** Liveliness, **(E)** Friendliness, and **(F)** Startle after music listening (AC or PM) in the different conditions (3D-SF or HD) compared with the level before it. **(G)** Felt unpleasantness of music rated after listening to music. Data are presented as means ± SEM; ***p < 0.001, **p < 0.01, *p < 0.05.

Investigating the effect of the presence of physical 3D-SF on moods when listening to music, we found that “Well-Being” was more strongly reduced after listening to both PM and AC in the 3D-SF condition than in the HD condition (Figure 4B).

The ANOVA revealed the following significant effects: a significant interaction of “sound reproduction condition” × “music” [*F*(1, 30) = 7.19, *p* = 0.012, partial η*^2^* = 0.19] and a significant main effect of “music” [*F*(1, 30) = 9.57, *p* = 0.0043, partial η*^2^* = 0.24] for Boredom (Figure 4A); a significant main effect of “sound reproduction condition” [*F*(1, 30) = 6.26, *p* = 0.018, partial η*^2^* = 0.17] for Well-Being (Figure 4B); no significant effects for Concentration (Figure 4C); a significant main effect of “music” [*F*(1,30) = 18.7, *p* = 0.0002, partial η*^2^* = 0.38] for Liveliness (Figure 4D); a significant main effect of “music” [*F*(1,30) = 10.29, *p* = 0.0032, partial η*^2^* = 0.26] for Friendliness (Figure 4E); a significant main effect of “music” [*F*(1, 30) = 20.87, *p* = 0.0001, partial η*^2^* = 0.41] for Startle (Figure 4F). A simple effects test demonstrated that Boredom for PM in the HD condition was significantly greater than that for AC in the HD condition, while no significant difference was observed in the 3D-SF condition (Figure 4A).

The effect of the presence of the physical 3D-SF was also found on emotions felt for the music. The unpleasantness felt for both PM and AC was larger in the 3D-SF condition than in the HD condition (Figure 4G). The felt pleasantness was considered to be not affected, because all the participants judged both AC and PM as “unpleasant” in both the 3D-SF and HD conditions. They only rated their felt unpleasantness, and their rate of pleasantness was automatically “1.” Although four participants in the HD condition experiments rated PM as 1 (= not unpleasant at all), their felt emotion was more unpleasant than pleasant for listening to PM in the HD condition. For the four participants, the degree of unpleasantness was quite small, namely close to 1. ANOVA was applied to the data of unpleasantness alone. The ANOVA demonstrated a significant main effect of “music” [*F*(1, 30) = 82.87, *p* < 0.0001, partial η*^2^* = 0.73] and “sound reproduction condition” [*F*(1, 30) = 8.35, *p* = 0.0071, partial η*^2^* = 0.22] (Figure 4G).

As a reference, the significance of the change in mood from the baseline in each condition is summarized in Table 1, for which a paired *t*-test between the value before and after listening to music was performed.

**TABLE 1:** Summary of comparison of each mood between, before, and after listening to music.

	Mood	Boredom		Well-Being		Concentration		Liveliness		Friendliness		Startle	
							
	Music	AC	PM	AC	PM	AC	PM	AC	PM	AC	PM	AC	PM
HD	*t*	2.04	6.28	7.15	7.01	0.37	2.03	0.22	2.82	1.77	1.84	6.91	3.88
	*p*	0.060	<0.0001	<0.0001	<0.0001	0.72	0.061	0.83	0.013	0.097	0.086	<0.0001	0.0015
	Cohen’s *d*	0.51	1.57	1.79	1.78	0.093	0.51	0.054	0.70	0.46	0.46	1.70	0.95
3D-SF	*t*	5.56	5.18	12.33	9.23	3.24	2.26	0.16	2.21	0.13	1.84	11.77	5.44
	*p*	<0.0001	0.0001	<0.0001	<0.0001	0.0055	0.039	0.87	0.043	0.9	0.085	<0.0001	<0.0001
	Cohen’s *d*	1.42	1.30	3.11	2.33	0.81	0.57	0.041	0.55	0.036	0.46	2.95	1.40

*The statistical significance was evaluated by a paired *t*-test.*

**t*, *t* value of paired *t*-test; *p*, *p* value of paired t-test; Cohen’s *d*, effect size of paired *t*-test.*

### Effect of Difference in Sound Reproduction Method on Change in Respiration Rate

**FIGURE 5:**
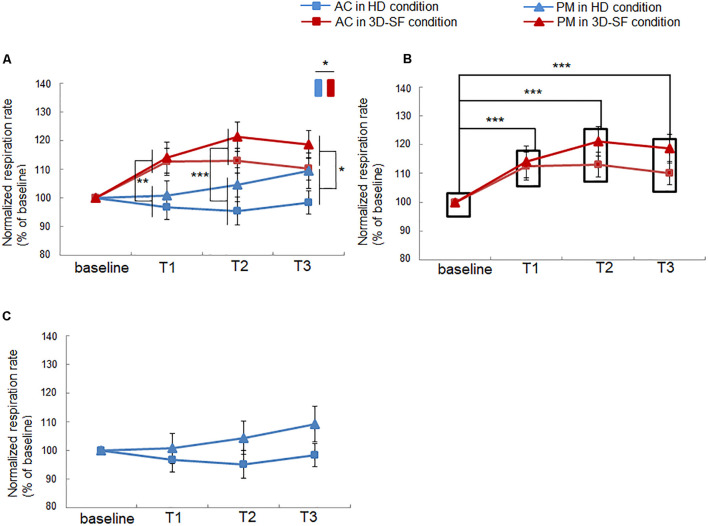
Effect of music listening on normalized respiration rate. **(A)** Comparison between the HD and 3D-SF conditions. **(B)** Comparison among the four time regions (baseline, T1, T2, and T3) in the 3D-SF condition. **(C)** Comparison among the four time regions (baseline, T1, T2, and T3) in the HD condition. Data are presented as means ± SEM; ***p < 0.001, **p < 0.01, *p < 0.05.

We found that the respiration rate of the participants was higher when they listened to both PM and AC in the 3D-SF condition than before they had listened (Figure 5B), whereas such a change was not observed in the HD condition (Figure 5C), suggesting that the presence of a physical 3D-SF can increase the respiration rate of listeners. The ANOVA revealed significant interactions of “sound reproduction condition” × “time” [*F*(3, 90) = 5.59, *p* = 0.0015, partial η*^2^* = 0.16] and “music” × “time” [*F*(3, 90) = 3.68, *p* = 0.015, partial η*^2^* = 0.11], and significant main effects of “sound reproduction condition” [*F*(1, 30) = 6.83, *p* = 0.014, partial η*^2^* = 0.19] and “time” [*F*(3, 90) = 6.82, *p* = 0.0003, partial η*^2^* = 0.19]. Concerning the difference between the reproduction conditions, a simple effects test demonstrated that the normalized respiration rate was larger for the 3D-SF condition than for the HD condition at the T1 [*F*(1, 120) = 8.73, *p* = 0.0038], T2 [*F*(1, 120) = 12.41, *p* = 0.0006], and T3 [*F*(1, 120) = 4.6, *p* = 0.034] regions (Figure 5A). Concerning the difference along the time region, a simple effects test demonstrated that there was a significant difference in the normalized respiration rate among the four time regions in the 3D-SF condition independent of the type of music [*F*(3, 90) = 11.44, *p* < 0.0001], in which Ryan’s method of adjusting the *P* value showed that the normalized respiration rate in every music region was larger than the baseline (*t* = 4.16, *p* = 0.0001 for baseline vs. T1; *t* = 5.36, *p* < 0.0001 for baseline vs. T2; *t* = 4.5, *p* < 0.0001 for baseline vs. T3) (Figure 5B), but that no change in the normalized respiration rate was observed in the HD condition (Figure 5C).

### Effect of Difference in Sound Reproduction Method on Change in Heart Rate and Heart Rate Variability

**FIGURE 6:**
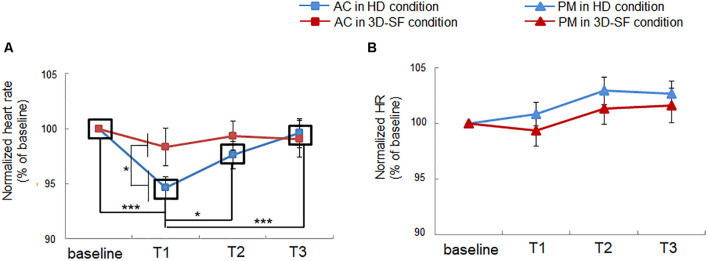
Effect of music listening on normalized heart rate. **(A)** Comparison between the HD and 3D-SF conditions for AC. **(B)** Comparison between the HD and 3D-SF conditions when listening to PM.

We showed a positive effect of the presence of the physical 3D-SF, but we also observed a negative side. Heart rate was reduced during listening to AC in the HD condition, whereas the reduction was diminished in the 3D-SF condition (Figure 6A). The ANOVA revealed significant interactions of “sound reproduction condition” × “music” × “time” [*F*(3, 90) = 3.16, *p* = 0.029, partial η*^2^* = 0.095] and “music” × “time” [*F*(3, 90) = 7.86, *p* = 0.0001, partial η*^2^* = 0.21] and significant main effects of “music” [*F*(1, 30) = 11.28, *p* = 0.0021, partial η*^2^* = 0.27], and “time” [*F*(3, 90) = 5.37, *p* = 0.0019, partial η*^2^* = 0.15]. Concerning the difference between the reproduction conditions, a simple effects test demonstrated that the normalized heart rate in AC listening was significantly lower for the HD condition than for the 3D-SF condition in the T2 region [*F*(1, 240) = 4.611, *p* = 0.033] (Figure 6A), while no significant difference between the 3D-SF and HD conditions was observed for PM (Figure 6B). Concerning the difference along the time region, a simple effects test demonstrated that there was a significant difference in the normalized heart rate among the four time regions for AC [*F*(3, 180) = 9.78, *p* < 0.0001], in which Ryan’s method of adjusting the *P* value showed that the normalized heart rate in T1 was lower than in the other regions [*t*(180) = 4.98, *p* < 0.0001 for T1 vs. baseline; *t*(180) = 2.55, *p* = 0.012 for T1 vs. T2; *t*(180) = 4.25, *p* < 0.0001 for T1 vs. T3] (Figure 6A).

In contrast to heart rate, neither a significant interaction nor a main effect was observed in HRV. The HRV data are summarized in Table 2.

**TABLE 2:** Summary of heart rate variability (HRV).

		lnLF			lnHF			lnLF/lnHF		
				
		T1	T2	T3	T1	T2	T3	T1	T2	T3
AC	HD	1.00 ± 0.04	1.01 ± 0.04	1.01 ± 0.04	1.04 ± 0.06	1.02 ± 0.05	0.99 ± 0.05	0.98 ± 0.06	1.00 ± 0.04	1.04 ± 0.04
	3D-SF	0.98 ± 0.03	0.98 ± 0.03	1.05 ± 0.04	1.01 ± 0.03	0.99 ± 0.03	1.00 ± 0.03	0.98 ± 0.03	1.00 ± 0.03	1.05 ± 0.04
PM	HD	1.00 ± 0.03	1.00 ± 0.03	1.04 ± 0.05	0.98 ± 0.03	0.97 ± 0.03	0.98 ± 0.04	1.03 ± 0.04	1.05 ± 0.04	1.07 ± 0.05
	3D-SF	1.00 ± 0.03	0.97 ± 0.03	1.00 ± 0.03	1.03 ± 0.04	1.03 ± 0.04	1.00 ± 0.04	0.98 ± 0.03	0.95 ± 0.04	1.01 ± 0.03

*All the data are normalized by dividing them by the corresponding baseline value. Data are presented as means ± SEM.*

## Discussion

This study examined differences in psychological and physiological responses between two reproduction conditions, namely the 3D-SF and HD conditions, when participants listened to music. The participants could experience spatial-audio information in the HD condition as rich as that in the 3D-SF condition, and the difference between the 3D-SF and HD conditions was the presence or absence of the physical 3D-SF. This suggests that our results can be explained by the effects of non-auditory perception, such as tactile perception. In the psychological responses, we analyzed “unpleasantness” as a parameter of the emotional response to music stimuli and “Well-Being,” “Boredom,” “Liveliness,” “Friendliness,” “Concentration,” and “Startle” as parameters of mood. We found a significant increase in unpleasantness. Although “Well-Being” showed a significantly larger decrease in the 3D-SF condition than in the HD-condition, none of the other moods showed significantly larger effects in the 3D-SF condition than in the HD-condition. These results suggest that a non-auditory cue provided by the presence of a 3D-SF facilitates emotions felt in response to music as well as changes in moods if listeners have low arousal and valence opposite to the felt emotion. In the physiological responses, we analyzed respiration rate, heart rate, and heart rate variability (HRV). We found that when the participants were in the 3D-SF condition, the respiration rate increased significantly during listening sessions regardless of the type of music, whereas no changes in HRV indices were observed in either the 3D-SF or HD conditions. Since an increase in the respiration rate is typically accompanied by an increase in sympathetic nerve activity (Bernardi et al., 2006; Salimpoor et al., 2011; Koelsch and Jancke, 2015), our finding of an increase in the respiration rate of the participants without any change in lnLF/lnHF when they listened to music is a novel one. Heart rate was reduced when they listened to AC in the HD condition, which could be regarded as an orienting response (Porges, 1995) induced by aversive stimuli (Bradley et al., 2001; Ooishi and Kashino, 2012). The reduction in heart rate was suppressed when they listened to AC in the 3D-SF condition. These results suggest that a non-auditory perception evoked by the presence of a 3D-SF activates the respiratory system without any effect on the sympathetic nervous system and, through the activation of the respiration system, suppresses the activity of neurons that evoke the aversive stimulus-induced orienting response.

The reproduction of a real sound field for listeners has been achieved in mainly two methodological ways. One is based on the wave field synthesis technique (Berkhout et al., 1993; Ise, 1999). The original sound is recorded with multichannel microphone arrays, and reproduced with multichannel speaker arrays. To enable the speaker arrays to reproduce the sound field with the best approximation, inverse filters for the multi-input/multi-output system are convolved with sound signals. With this technique, listeners can experience as if they are surrounded with the original sound field. The other is based on the binaural reproduction technique (Paul, 2009). Contrary to listening through headphones to sound that is stereophonically recorded with two-channel microphones place at a chosen position in the sound field, binaurally recorded sound through headphones enables listeners to experience three-dimensional auditory perception. Binaural recording is usually performed with a dummy head that has two microphones at the position of the ear drums.

Listening to sound within a sound field reproduced with the wave field synthesis technique and listening to binaurally recorded sound through headphones provide almost the same spatial-audio information as that in the original sound field. However, sound produced by the wave field synthesis technique has a physical sound field, whereas binaurally recorded sound has none. As reported in earlier studies (Boone et al., 1995; Theile and Wittek, 2004), sound field synthesis enables listeners to feel as if they are surrounded by sound. We assume that this feeling is due to not only auditory perception but also other channels of perception, such as tactile ones. Another earlier study showed the effect of the “Music Bath,” which can relieve pain or anxiety by presenting sounds from loudspeakers as a source of vibration (Skille, 1987a,b). This is consistent with our assumption in the sense that the effects of sound other than its auditory perception affect listeners. For example, we found that the felt unpleasantness was larger when the participants listened to music in the 3D-SF condition than in the HD condition, suggesting that non-auditory perception induced by the presence of a physical 3D-SF enhances the impression of music for listeners.

Terasaki et al. (1992) indicated that “Boredom,” “Well-Being,” “Concentration,” “Liveliness,” “Friendliness,” and “Startle,” the parameters used in this study, correspond to fatigue, well-being, concentration or anxiety, energy, social affection, and anxiety, respectively, as defined by Lebo and Nesselroade (1978). We found that the decrease in “Well-Being” was larger in the 3D-SF condition than in the HD condition after the participants had listened to music (Figure 4B). Considering this together with the results of unpleasantness, and considering that “Well-Being” (Levo’s well-being) is similar to relaxation, it is suggested that unpleasant music reduced the relaxation level of the participants. Interestingly, the degree of unpleasantness does not determine the size of the reduction of relaxation, because the unpleasantness for AC was higher than that for PM (Figure 4G), while the size of the reduction of Well-Being was not significantly different between AC and PM (Figure 4B). Our results suggest that not the degree of unpleasantness itself but the increase in unpleasantness caused by the presence of physical 3D-SF boosts the reduction of relaxation level. In contrast to “Well-Being,” “Liveliness” was not affected by the difference in the reproduction condition (Figure 4D). Considering that “Liveliness” (Lebo’s energy) is similar to excitement, the difference between “Well-Being” and “Liveliness” is the degree of arousal; arousal is low for “Well-Being,” while it is high for “Liveliness” (Russell et al., 1989). Therefore, it is suggested that moods with low arousal could be affected by the presence of a physical 3D-SF. Actually, “Friendliness” (Lebo’s social affection, similar to love) and “Startle” (Lebo’s anxiety) are categorized as high arousal (Russell et al., 1989). Although Lebo’s concentration was not categorized by Russel, the “Concentration” we used corresponds to Lebo’s anxiety as well as to Lebo’s concentration, meaning that “Concentration” could also be categorized as high arousal (Russell et al., 1989). However, our assumption does not consider “Boredom” (Lebo’s fatigue), because it is categorized as low arousal (Russell et al., 1989). The difference between “Well-Being” and “Boredom” is the degree of valence. “Well-Being” (similar to relaxation) is categorized as high valence, while “Boredom” is categorized as low valence (Russell et al., 1989). Since the music stimuli in this study were rated as unpleasant by all the participants, the non-auditory perception induced by the presence of the physical 3D-SF could have facilitated their moods if the moods were categorized as having low arousal and valence opposite to the emotion felt in response to music stimuli. We assume that the presence of a physical 3D-SF could facilitate Boredom when listening to music rated as pleasant.

The impact of the presence of the physical sound field on physiological activity when listening to music was also detected in this study. We observed a significant increase in respiration rate in the 3D-SF condition, regardless of the type of music (Figure 5). Previous studies have demonstrated the contribution of music features, such as tempo, to the modulation of respiration. In particular, the entrainment of respiration rate to music tempo has been frequently discussed (Haas et al., 1986; Bernardi et al., 2006; Etzel et al., 2006; Khalfa et al., 2008). Our results could be considered as novel findings in the sense that the response of respiration to music stimuli in our study was inconsistent with earlier studies. The increase in respiration rate during music listening is typically induced together with an increase in the sympathetic nerve activity (Bernardi et al., 2006; Salimpoor et al., 2011; Koelsch and Jancke, 2015), whereas our results showed no significant change in lnLF/lnHF with listening to both AC and PM in both the 3D-SF and HD conditions (Table 2). The LF/HF ratio of HRV is an index of the activities of neurons in the rostral ventrolateral medulla (RVLM) (Thomas and Spyer, 1999; Dun et al., 2000; Jiang et al., 2011; Shahid et al., 2012; Chen et al., 2013), one of the primary regulators of the sympathetic nervous system that controls cardiac activity (Shields, 1993; Kumagai et al., 2012), and is often used to represent music listening-induced excitation (Bernardi et al., 2006; Ooishi et al., 2017). Taking our results together with those of previous studies, we believe that the presence of the physical 3D-SF enhanced respiration rate during music listening through channels other than auditory perception without any effect on sympathetic cardiac control.

Interestingly, heart rate was reduced with listening to AC in the HD condition, which suggests a contribution of parasympathetic nerve activity. The nucleus ambiguus (NA) is one of the primary regulators of the parasympathetic nervous system that controls cardiac activity (Porges, 1995). The HF component of HRV can be an index of parasympathetic nerve activity originating from the NA and is often used to represent music-induced relaxation (Zhou et al., 2010; Ooishi et al., 2017). Therefore, no change in the HF component means no change in the cardiac parasympathetic modulation originating from the NA. In the literature, Porges proposed in his earlier study that there is a polyvagal mechanism that contains two types of nuclei inducing heart rate deceleration (Porges, 1995). One of the nuclei is the NA noted above, and the other is the dorsal motor nucleus of the vagus (DMNX). We assume that the heart rate deceleration that occurred while the participants were listening to AC in the HD condition originated from activation of the DMNX, which is categorized as an orienting response (Porges, 1995). Heart rate deceleration as an orienting response is frequently induced by aversive sensory stimuli (Bradley et al., 2001; Ooishi and Kashino, 2012). However, heart rate did not change with listening to AC in the 3D-SF condition, in which the participants rated it more unpleasant than in the HD condition (Figure 4G). We propose that the enhancer of respiration rate might suppress the activity of neurons in the DMNX. Actually, there is a connection between respiratory systems and the DMNX. For example, the DMNX receives input from the nucleus tractus solitarius (Kalia, 1981).

In conclusion, we found that the presence of a physical sound field when listening to music facilitates psychological and physiological responses. Music listening in the 3D-SF condition enhanced the decrease in Well-Being and the felt unpleasantness for both AC and PM compared with in the HD condition. Respiration rate was increased compared with the baseline only with music listening in the 3D-SF condition. Since reproduced music in both the 3D-SF and HD conditions has spatial-audio information as rich as that of the original one, we suggest that the presence of the physical sound field affects listeners through channels other than auditory perception, such as tactile ones, when listening to music.

### Limitations

Before starting this study, we prepared AC as the representative of unpleasant (e.g., feeling of fear or a thrill) but interesting music and PM as that of pleasant or impressive music. We hypothesized that the felt emotion for AC and PM would be larger when listening to them with a high-quality3D-SF reproduction system compared with listening to the same pieces binaurally recorded with a dummy head and reproduced with headphones. Our hypothesis was based on the possibility that the physical 3D-SF enhances the psychological and physiological responses of the listeners through channels other than auditory perception, such as tactile ones. Actually, we (the four authors of this study) regarded AC as extremely unpleasant music that could raise goose bumps due to aversion and PM as very pleasant and impressive, and we felt that the degree of such emotions was larger when listening to music in the 3D-SF condition than in the HD condition. However, all the students (six men, age 22∼24) from Meiji University recruited as participants in the preliminary experiment assessed PM as unpleasant. In particular, the unpleasantness of PM increased when they listened to it in the 3D-SF condition. In the experiments of this study, as well as in the preliminary experiment, all the participants judged both AC and PM in both the 3D-SF and HD conditions as unpleasant. We, therefore, asked the participants to rate the degree of felt unpleasantness without pleasantness when they listened to the music. Their rate of pleasantness was automatically “1.” Therefore, we performed ANOVA only on the data of unpleasantness. Our results demonstrated that the unpleasantness of both AC and PM in the 3D-SF condition was larger than in the HD condition. A future direction will include examining the effect of the presence of the 3D-SF when participants listen to music that they appreciate as pleasant.

Among the 32 participants recruited for this study, there were only 3 females, so there may have been biases in how they responded to the music. An earlier study demonstrated that emotional and psychophysiological responses to music are larger for females than for males (Gupta and Gupta, 2016). In addition, all the participants were non-musicians. Differences in responses to music have also been observed between musicians and non-musicians. For example, musicians have greater respiratory sensitivity to tempo than non-musicians (Bernardi et al., 2006). A future direction will include considering the balance of genders (male and female) or music-training histories (musicians and non-musicians) to examine the differences or similarities between them (male vs. female, musicians vs. non-musicians).

We used a dummy head to perform binaural recording of music because it enables listeners to perceive spatial-audio information as rich as that in the original sounds due to its inclusion the effect of the head-related transfer function (HRTF) (Paul, 2009). Strictly speaking, the HRTF is different between individuals because of differences in the shape of the head, ear pinna, body, and features of clothes they wear (Riederer, 2003; Wersényi and Illényi, 2005). However, the effect of binaurally recorded sounds with a dummy head reproduced through headphones is sufficiently larger than it is for normal two-channel stereophonic sounds through headphones (Paul, 2009). Currently it is too difficult to calculate individual HRTFs in each experiment, but we hope for a technology that will make it possible in the future.

In the HD condition, we re-recorded the reproduced music stimuli in the 3D-SF condition with the dummy head, although it would have been better to record the music pieces played live with the dummy head. The possible loss of sound information by re-recording could have affected the results of this study. The reason we performed the re-recording was to ensure that there would be no difference in the direction of the sound source between conditions. If the music pieces had been recorded with the fullerene-type microphone array and the dummy head simultaneously, the direction of the sound source would have differed between the respectively recorded sounds, because the position of the microphone array and the dummy head was different. To reduce the effect of re-recording, we set the sampling rate at 48 kHz and performed 24-bit quantization, which can sufficiently cover the human audible range, when recording music pieces with both the fullerene-type microphone array and the dummy head.

In this study, we proposed that the presence of a physical 3D-SF affects listeners through non-auditory perception. Since all the experiments were performed in a dark reproduction room, the contribution of vision could be ignored when considering the effect of the 3D-SF. Therefore, we predicted that tactile perception would be a possible source of the effect of the3D-SF; however, none of the data we obtained could prove this prediction. Furthermore, we asked the participants to keep their eyes open and to steadily sit on a chair and avoid bodily movement, including the head, as much as they could, but, of course, they might not have been able to keep their eyes open during the entire listening session (excluding eye blinks) or achieve complete stillness. Closing the eyes while listening to negative music has been shown to produce greater ratings of emotionality than opening them (Lerner et al., 2009), and head movement affects spatial-audio perception (Kondo et al., 2012). Therefore, failure to keep the eyes open the whole time and the lack of head fixation might weaken the possibility of the contribution of non-auditory perception, such as tactile to the effect of music listening in the presence of a physical 3D-SF. Future studies should include an attempt to directly reveal that tactile perception contributes to the effect of music listening in the presence of a physical 3D-SF on listeners.

## Data Availability Statement

The raw data supporting the conclusions of this article will be made available by the authors, without undue reservation.

## Ethics Statement

The studies involving human participants were reviewed and approved by the Ethics and Safety Committees of Meiji University. The patients/participants provided their written informed consent to participate in this study.

## Author Contributions

YO, MKo, and KU designed the psychological experiments. MKo collected the autonomic response data and self-reported ratings, and prepared the experimental speech stimuli. YO analyzed the autonomic responses data and self-reported ratings, and evaluated the results. KU provided the three-dimensional sound reproduction system. YO, MKo, and MKa wrote the manuscript. All authors contributed to the article and approved the submitted version.

## Conflict of Interest

YO and MKa are employees of NTT Communication Science Laboratories, which is a basic-science research section of Nippon Telegraph and Telephone (NTT) Corporation. There are no pending patents involving the reported research. There are no products in development or marketed products to declare. Thus, the authors adhere to the policies of Frontiers. The remaining authors declare that the research was conducted in the absence of any commercial or financial relationships that could be construed as a potential conflict of interest.

## Publisher’s Note

All claims expressed in this article are solely those of the authors and do not necessarily represent those of their affiliated organizations, or those of the publisher, the editors and the reviewers. Any product that may be evaluated in this article, or claim that may be made by its manufacturer, is not guaranteed or endorsed by the publisher.

## Footnotes

1 The pipe organ player kindly participated in this study on condition that we do not mention his/her name, and that the recording would be performed for only this tune.

2 The piece is commercially available; it was not composed for the purpose of our research. The composer gave us permission to use the piece on condition that we do not mention its title or the name of the composer, and that only this piece would be used. The composer played this piece live so we could record it with a multichannel microphone array.

## Abbreviations

BoSC: boundary surface control
3D: three-dimensional
HRV: heart rate variability
HF: high frequency
LF: low frequency
AC: acousmonium
PM: pipe organ music
RVLM: rostral ventrolateral medulla
NA: nucleus ambiguus
DCMX: dorsal motor nucleus of the vagus.
