# Supplementary material for: Presence of Three-Dimensional Sound Field Facilitates Listeners’ Mood, Felt Emotion, and Respiration Rate When Listening to Music
Source: Front Psychol. 2021 Nov 17;12:650777. doi: 10.3389/fpsyg.2021.650777 (PMC8637927; doi:10.3389/fpsyg.2021.650777)
Supplement: Supplementary file 1 [file Presentation_1.pdf]

Fig. S1

A

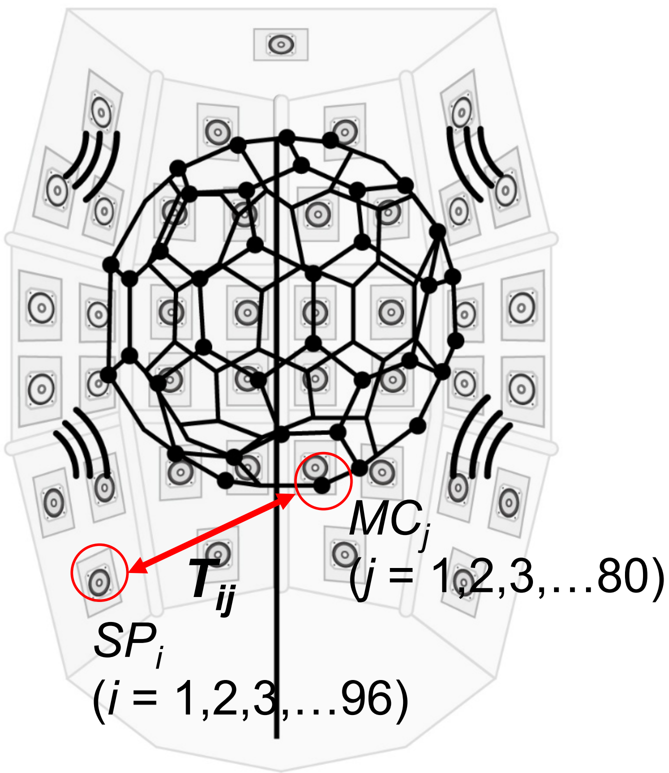

Type-1 inverse filters: invT_{ij}
(T_{ij} + invT_{ij} = 0)

B

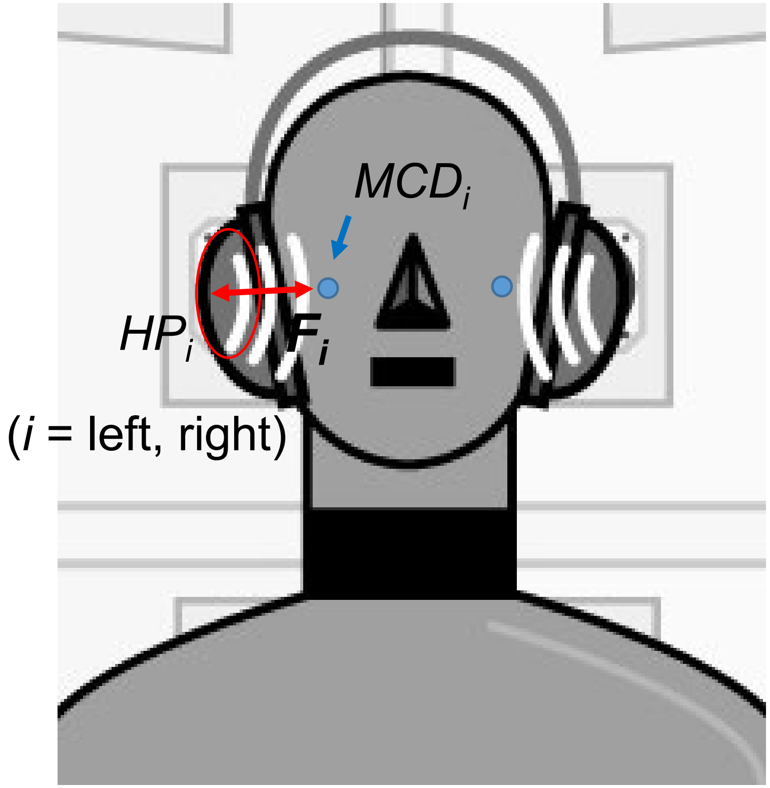

Type-2 inverse filters: invF_i
(F_i + invF_i = 0)

Supplementary Figures

Fig. S1. Inverse filters were prepared to make experimental music stimuli. *(A)* To achieve complete reproduction of the original sound field in the reproduced area, time stretched pulse (TSP) signals were reproduced from speakers in the reproduction room and recorded with the microphone array located on the listening position. By doing this process, transfer function T_{ij} between each speaker SP_i (i = 1,2,3,…96) and each microphone MC_j (j = 1,2,3,…80) was calculated. The inverse filters incorporated the inverse transfer function $invT_{ij}$, where $T_{ij} + invT_{ij} = 0$. These inverse filters , referred to as type 1, were convolved with the recorded music to make music stimuli in the 3D-SF condition. *(B)* To remove the frequency characteristics of headphones, TSP signals were reproduced through headphones and recorded with the microphones embedded in the dummy head. By doing this process, transfer function F_i between each headphone HP_i and each microphone in the dummy head MCD_i (i = left or right) was calculated. The inverse filters, referred to as type 2, consisted of the inverse function $invF_i$, where $F_i + invF_i = 0$. . The type-2 inverse filters were convolved with the music stimuli used in 3D-SF condition to make music stimuli in HD condition.
